# Evaluation of immunological, inflammatory, and oxidative stress biomarkers in gasoline station attendants

**DOI:** 10.1186/s40360-019-0355-1

**Published:** 2019-12-19

**Authors:** Angela Maria Moro, Elisa Sauer, Natália Brucker, Mariele Feiffer Charão, Bruna Gauer, Sabrina Nunes do Nascimento, Gabriela Goethel, Marta Maria Medeiros Frescura Duarte, Solange Cristina Garcia

**Affiliations:** 1 Laboratory of Toxicology (LATOX), Department of Analysis, Pharmacy Faculty, Federal University of Rio Grande do Sul, Porto Alegre, RS Brazil; 2 Specialized Faculty in the Health Area of Rio Grande do Sul (FASURGS), Passo Fundo, RS Brazil; 3 Post-graduate Program in Pharmaceutical Sciences, Federal University of Rio Grande do Sul, Avenida Ipiranga 2752, Santa Cecília, Porto Alegre, RS CEP: 90610-000 Brazil; 4 Department of Physiology and Pharmacology, Federal University of Santa Maria, Santa Maria, RS Brazil; 5 Health Sciences Institute, Feevale University, Novo Hamburgo, RS Brazil; 6 Department of Health Sciences, Lutheran University of Brazil, Santa Maria, RS Brazil

**Keywords:** Occupational toxicology, Benzene, Toluene, Xylene, CD80, CD86, Pro-inflammatory cytokines, Anti-inflammatory cytokines, Protein carbonyl, Glutathione S-transferase

## Abstract

**Background:**

Gasoline is a complex mixture of saturated and unsaturated hydrocarbons, in which aromatic compounds, such as BTX (benzene, toluene, and xylene) feature as the main constituents. Simultaneous exposure to these aromatic hydrocarbons causes a significant impact on benzene toxicity. In order to detect early alterations caused in gasoline station attendants exposed to BTX compounds, immunological, inflammatory, and oxidative stress biomarkers were evaluated.

**Methods:**

A total of 66 male subjects participated in this study. The gasoline station attendants (GSA) group consisted of 38 gasoline station attendants from Rio Grande do Sul, Brazil. The non-exposed group consisted of 28 subjects who were non-smokers and who had no history of occupational exposure. Environmental and biological monitoring of BTX exposure was performed using blood and urine.

**Results:**

The GSA group showed increased BTX concentrations in relation to the non-exposed group (*p* < 0.001). The GSA group showed elevated protein carbonyl (PCO) levels and pro-inflammatory cytokines, decreased expression of CD80 and CD86 in monocytes, and reduced glutathione S-transferase (GST) activity compared to the non-exposed group (*p* < 0.05). BTX levels and *trans,trans*-muconic acid levels were positively correlated with pro-inflammatory cytokines and negatively correlated with interleukin-10 contents (*p* < 0.001). Increased levels of pro-inflammatory cytokines were accompanied by increased PCO contents and decreased GST activity (*p* < 0.001). Furthermore, according to the multiple linear regression analysis, benzene exposure was the only factor that significantly contributed to the increased pro-inflammatory cytokines (*p* < 0.05).

**Conclusions:**

Taken together, these findings show the influence of exposure to BTX compounds, especially benzene, on the immunological, inflammatory, and oxidative stress biomarkers evaluated. Furthermore, the data suggest the relationship among the evaluated biomarkers of effect, which could contribute to providing early signs of damage to biomolecules in subjects occupationally exposed to BTX compounds.

## Background

Gasoline station attendants are an important group at occupational risk to BTX (benzene, toluene, and xylene) compounds [1–3]. Among the constituents of gasoline, benzene stands out for its hazardous effects on human health [4–6]. Furthermore, simultaneous exposure to benzene and other aromatic hydrocarbons, such as toluene and xylene, contributes to maximizing benzene toxicity [7].

Benzene exposure in occupational settings has been progressively decreased as a result of preventive actions [8]. However, there are health concerns for workers related to low levels of occupational exposure. These concerns are linked to the fact that benzene is a well-recognized genotoxic human carcinogen, classified as a Group I chemical by the International Agency for Research on Cancer, and without any known threshold dose [3].

In addition, it is well known that the hematologic disorders, leukemia and myelodysplastic syndrome are caused by occupational benzene exposure, as well as various deleterious effects on many other biological systems after long-term exposures [9, 10]. Epidemiological and experimental studies have shown that benzene exposure can lead to non-cancer health effects, such as, genotoxicity, hematotoxicity, hepatotoxicity and nephrotoxicity [2, 11, 12]. The mechanisms of benzene toxicity remain elusive. However, it is well-known that reactive oxygen species (superoxide anion, hydrogen peroxide, hydroxyl radical) resulting from benzene metabolism may damage biomolecules, inducing oxidative stress [10, 11].

Biomonitoring is a mandatory health protection measure for workers occupationally exposed to benzene [13, 14]. Biological monitoring has been used as a potential tool for better assessing integrated benzene exposures and may contribute to the diagnosis and treatment of occupational diseases [15]. Since there are no safe limits for exposures to carcinogens such as benzene, simultaneous evaluation of biomarkers of exposure and effect may be useful in the estimation and reduction of risks caused by occupational exposure to this xenobiotic. Such assessments may also be adopted for preventative health initiatives for improving the safety of occupationally exposed workers [9, 16].

A number of previous studies by our research group were conducted with the aim of establishing new biomarkers of early damage, which might be helpful in biomonitoring actions related to occupational exposure to benzene. Many early biomarkers of genetic [11], hematological [4], renal, hepatic [2], and immunological [2, 17] alterations, caused by low levels of occupational benzene exposure, were proposed for the continuous monitoring of occupational benzene hazards. Furthermore, these findings may be key elements in the elucidation of different benzene toxicity mechanisms.

Based on immunological biomarkers previously proposed by Moro et al. [4], the work conducted by Sauer et al. [17] showed possible immunotoxicity and carcinogenicity mechanisms arising from benzene exposure. Furthermore, alterations in inflammation biomarkers were also reported by Sauer et al. [17]. In order to continue the previous studies of our research group, this paper aimed to evaluate immunological, inflammatory, and oxidative stress biomarkers in gasoline station attendants, and verify the possible influence of benzene exposure on these biomarkers of effect.

## Methods

### Study population

Sixty-six individuals were enrolled in this study. The exposed group consisted of 38 gasoline station attendants (GSA) from Rio Grande do Sul, Brazil. All subjects had been working in their current job position for at least 6 months. The non-exposed (NE) group consisted of 28 subjects who were non-smokers and who had no history of occupational exposure to benzene or other xenobiotics. Each participant was interviewed about aspects of general health, lifestyle, smoking status, and history of exposure.

The average ages of the GSA and control groups were 32.1 ± 1.7 years and 30.4 ± 1.8 years, respectively. No significant difference between the GSA and control groups was found related to age. The mean exposure time in the GSA group was 8.5 ± 1.6 years (range: 0.5–32 years).

This study was approved by the research ethics committee of the Federal University of Rio Grande do Sul/RS (No. 21728/11) and written informed consent was obtained from all participants prior to their enrollment in the study.

### Sample collection

The sampling was conducted at the end of the work shift after 3–4 consecutive days of exposure. Personal monitors were used to assess airborne BTX concentration during the daily work shift, for approximately 8 h. After air sampling, the samplers were stored at − 20 °C until analysis. Urine samples were collected for benzene, toluene, and xylene metabolites and creatinine determination. The samples were stored in polyethylene bottles at − 80 °C until further analysis. Venous blood samples were collected by venipuncture using vacuum tubes. EDTA-blood tubes were collected and centrifuged at 1500 g for 10 min at 4 °C. Aliquots of EDTA-plasma were stored at − 80 °C until analysis of the protein carbonyl content. A blood heparin tube was collected for glutathione S-transferase enzymatic activity determination and co-stimulatory molecules of CD80 e and CD86 assay analysis. A vacuum blood tube without anticoagulant was centrifuged at 1500 g for 10 min at room temperature. The serum obtained was aliquoted and stored at − 80 °C until inflammatory cytokine analysis.

### Exposure assessment

Personal breathing-zone air samples were collected using passive samplers (SKC 575–002®). BTX (benzene, toluene, *o-, m-, p-*xylene) were desorbed with dichloromethane and analyzed by gas chromatography and flame ionization detection (GC-FID; PerkinElmer, USA). GC column Innowax (25 m, 0.2 mm, 0.4 μm) was used. The initial oven temperature was 40 °C, and the temperature was increased by 4 °C min^− 1^ until it reached 53 °C, followed by an oven ramp rate of 40 °C min^− 1^ up to 200 °C. The FID detector temperature was kept at 250 °C.

Quantification of urinary benzene metabolite (*trans,trans*-muconic acid - *t,t*-MA) was carried out by high-performance liquid chromatography with UV detection (HPLC-UV; Shimadzu, USA) after solid phase extraction (SPE) according to a previously described analytical method [18], with modifications as previously described [4]. Quantification of urinary metabolites of toluene (hippuric acid - HA) and xylene (methylhippuric acid - mHA) were simultaneously performed by HPLC-UV [19]. The creatinine concentration was measured by spectrophotometry as previously described [20] using commercial kits (Doles reagents, Brazil).

### Immunological biomarkers

CD80 and CD86 expression by monocytes was analyzed by flow cytometry. The samples were processed within 24 h. Erythrocytes lysis was performed using an ammonium chloride solution (0.13 M) and the leukocytes were resuspended with PBS buffer. 10^6^ leukocytes were incubated with PE-conjugated anti-CD80 and FITC-conjugated anti-CD86. The antibodies were diluted 1:100 with PBS and incubated in the dark at 4 °C for 20 min. Cells were analyzed by FACSC Canto II Flow Cytometer (Becton Dickinson, San Jose, CA) with Flow Jo Software (TreeStar). Monocyte cells were identified by manual gating according to side scatter and size.

### Inflammatory biomarkers

Interleukin-1β (IL-1β), interleukin-6 (IL-6), interleukin-10 (IL-10), tumor necrosis factor-α (TNF-α), and interferon-γ (IFN-γ) were analyzed using ELISA methods according to the manufacturer’s instructions (Quantikine human immunoassays, R&D Systems).

### Oxidative stress biomarkers

Protein carbonyl (PCO) levels were determined using a noncompetitive ELISA method in accordance with Buss et al. [21] with some modifications. Total protein concentration in plasma was measured by the Bradford method using bovine serum albumin as standard. PCO levels were determined as follows: plasma samples were diluted with PBS buffer to a normalized concentration of 4 mg protein mL^− 1^ and then samples were derivatized with 2,4-dinitro-phenylhydrazine (DNPH) and incubated in Maxisorb multiwallplates (Nunc Immuno 96 MicrowellTM Maxisorp) overnight at 4 °C in the dark. Protein carbonyls were detected using a dinitrophenyl rabbit IgG-antiserum (Sigma) as the primary antibody and a monoclonal anti-rabbit immunoglobulin G peroxidase conjugate (Sigma) as the secondary antibody. Color development was performed with o-phenylenediamine and H_2_O_2_ and the reaction was stopped with H_2_SO_4_ after 15 min incubation at 37 °C. The absorbance was measured using a microplate reader with a detection wavelength of 492 nm.

Glutathione S-transferase (GST) activity was performed in 96-well microplates and was read in a microplate reader (SpectraMax M2e, Molecular Devices), in accordance with Habig et al. [22]. GST was determined using CDNB (1-chloro-2,4-dinitrobenzene) as substrate and 0.15 M GSH.

### Statistical analysis

The data analysis was performed using the IBM SPSS Statistics software (version 19). All study variables were tested for normality using the Shapiro-Wilk test. Comparisons between groups were obtained using Student’s t-test and the Mann-Whitney U-test. The results were expressed as mean ± SEM or median (interquartile range), according to variable distribution. Correlation tests were performed using Pearson’s correlation coefficient and Spearman’s rank according to each variable. Multiple linear regression models were applied to verify the relation between BTX exposure, immunological biomarkers, inflammatory biomarkers, and oxidative stress biomarkers. The significance level for all tests was *p* < 0.05.

## Results

The data on environmental BTX exposure levels are reported in Table 1. The GSA group was exposed to significantly higher BTX concentrations than the non-exposed group (*p* < 0.001). Additionally, the data showed that the GSA group was exposed to very low levels of BTX, with all values found to be below the ACGIH (American Conference of Governmental Industrial Hygienists) limits.

**Table 1:** Environmental and biological monitoring

	Non-exposed group (*n* = 28)	GSA group (*n* = 38)	ACGIH limits
Benzene (μg m^− 3^)	43.3 (35.3–63.3)	105.9 (63.3–216.8)^a^	1600.0
Toluene (μg m^− 3^)	107.4 (85.9–201.3)	311.5 (207.7–578.2)^a^	75,260.0
*o-*Xylene (μg m^− 3^)	37.5 (23.0–224.4)	59.4 (29.5–433.2)^a^	
*m-*Xylene (μg m^− 3^)	20.1 (17.4–25.6)	32.5 (27.8–37.4)^a^	433,540.0*
*p-*Xylene (μg m^− 3^)	21.2 (18.4–27.0)	31.0 (22.2–34.6)^a^	
*t,t*-MA (μg g^− 1^ creatinine)	74.8 (47.6–121.5)	421.9 (292.2–694.6)^a^	500.0
HA (g g^− 1^ creatinine)	0.3 (0.2–0.4)	0.3 (0.2–0.6)	1.6
m-HA (g g^− 1^ creatinine)	n.d.	n.d.	1.5

The values are expressed as median (interquartile range)

*t,t-MA trans,trans*-muconic acid, *HA* hippuric acid, *mHA* methylhippuric acid, *n.d.* not detectable

*total value to the sum of xylene isomers

^a^*p* < 0.001

Higher *t,t*-MA levels were found for the GSA group when compared to the non-exposed group (*p* < 0.001; Table 1). Although the *t,t*-MA levels were higher in the GSA group compared to the non-exposed group, the median values were below the biological exposure index (BEI: 500 μg g^− 1^ creatinine) established by the ACGIH.

The urinary biomarker of exposure to toluene was not significantly changed between the groups (*p* > 0.05; Table 1). HA median values were below the biological limit value for occupationally exposed people (BEI: 1.6 g g^− 1^ creatinine; ACGIH). Furthermore, the urinary biomarker of exposure to xylene could not be detected in any members of the study population (Table 1).

**Fig. 1:**
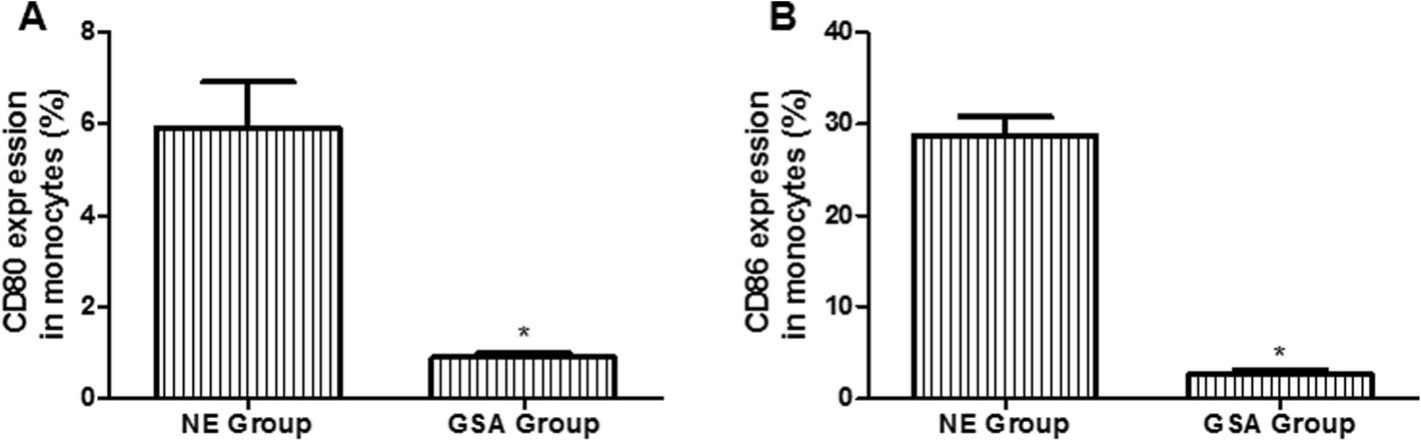
Immunological biomarkers in GSA group (*n* = 38) and non-exposed group (*n* = 28). **a** CD80 expression in monocytes. **b** CD86 expression in monocytes. Data are expressed as mean ± SEM. *p < 0.001

Figure 1 shows the immunological biomarker results. Decreased expression of CD80 (Fig. 1a) and CD86 (Fig. 1b) was observed in the membrane of monocytes of the GSA group compared to the non-exposed group (*p* < 0.001).

**Fig. 2:**
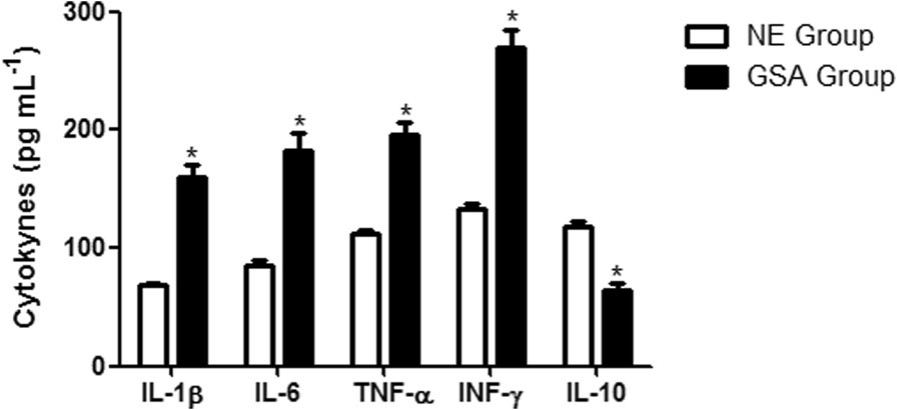
Inflammatory biomarkers in GSA group (*n* = 38) and non-exposed group (*n* = 28). Data are expressed as mean ± SEM. *p < 0.001

The cytokines levels are shown in Fig. 2. The GSA group showed increased levels of pro-inflammatory IL-1β, IL-6, TNF-α, and IFN-γ in relation to the non-exposed group (*p* < 0.001). In addition, reduced levels of anti-inflammatory IL-10 was observed in the GSA group compared to the non-exposed group (*p* < 0.001).

**Fig. 3:**
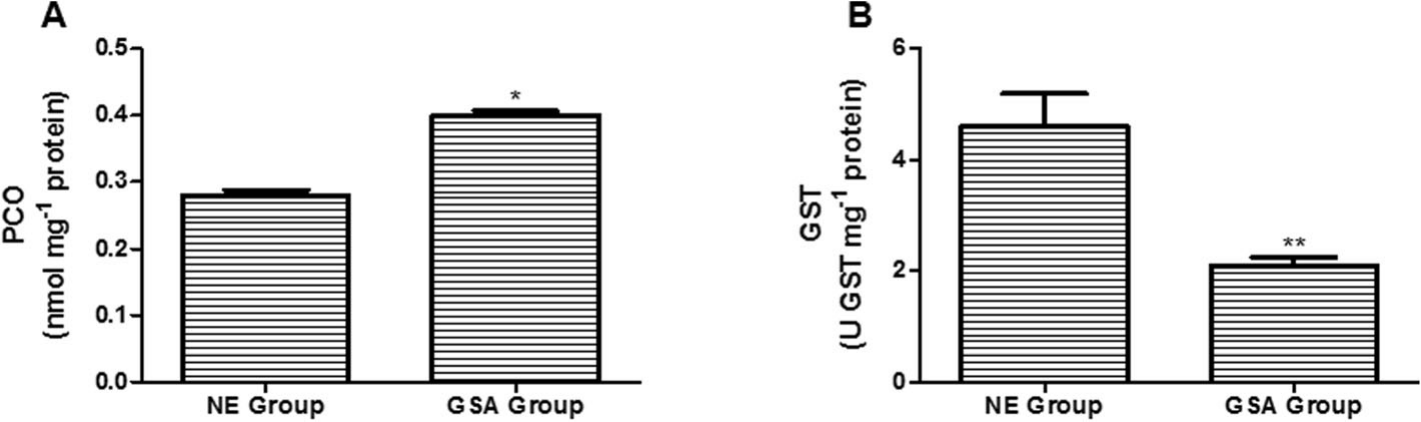
Oxidative stress biomarkers in GSA group (*n* = 38) and non-exposed group (*n* = 28). **a** Protein carbonyl (PCO) levels. **b** Glutathione S-transferase (GST) activity. Data are expressed as mean ± SEM. *p < 0.001. **p < 0.05

In relation to oxidative stress biomarkers, the protein damage was assessed through PCO levels (Fig. 3a). The GSA group showed an elevated PCO concentration compared to the non-exposed group (*p* < 0.001). The antioxidant status was evaluated through GST enzymatic activity (Fig. 3b). Decreased GST activity was observed in the GSA group compared to the non-exposed group (*p* < 0.05).

Univariate correlations were performed in all subjects, as a single sample. Strong correlations were found among the BTX organic solvents present in the gas station environment. Personal benzene exposure was positively correlated with airborne toluene concentration (*r* = 0.70; *p* < 0.001), *o-*xylene (*r* = 0.40; *p* < 0.05), *m-*xylene (*r* = 0.80; *p* < 0.001), and *p-*xylene (*r* = 0.73; *p* < 0.001) levels. Additionally, personal toluene exposure was also correlated with *o-*xylene (*r* = 0.40; *p* < 0.05), *m-*xylene (*r* = 0.75; *p* < 0.001), and *p-*xylene (*r* = 0.64; *p* < 0.001) concentrations.

Also, the increased levels of personal benzene exposure was accompanied by urinary *t,t*-MA excretion (*r* = 0.35; *p* < 0.01). Environmental toluene and xylene levels were not significantly correlated with their urinary biomarkers (*p* > 0.05).

Environmental BTX levels and *t,t*-MA concentrations were significantly correlated with immunological, inflammatory, and oxidative stress biomarkers (Table 2).

**Table 2:** Spearman’s correlations among personal BTX exposure, *trans,trans*-muconic acid, immunological, inflammatory, and oxidative stress biomarkers (*n* = 66)

	Benzene (μg m^− 3^)	Toluene (μg m^− 3^)	*o-*Xylene (μg m^− 3^)	*m-*Xylene (μg m^− 3^)	*p-*Xylene (μg m^− 3^)	*t,t*-MA (μg g^− 1^ creatinine)
CD80 monocyte expression (%)	*r* = − 0.26 (*p* < 0.05)	*r* = − 0.32 (*p* < 0.05)	*p* > 0.05	*p* > 0.05	*p* > 0.05	*r* = − 0.43 (*p* < 0.001)
CD86 monocyte expression (%)	*r* = − 0.46 (*p* < 0.001)	*r* = − 0.58 (*p* < 0.001)	*r* = − 0.43 (*p* < 0.05)	*r* = − 0.41 (*p* < 0.05)	*r* = − 0.43 (*p* < 0.05)	*r* = − 0.47 (*p* < 0.001)
IL-1β (pg mL^− 1^)	*r* = 0.43 (*p* < 0.001)	*r* = 0.37 (*p* < 0.05)	*p* > 0.05	*r* = 0.46 (*p* < 0.05)	*r* = 0.39 (*p* < 0.05)	*r* = 0.54 (*p* < 0.001)
IL-6 (pg mL^− 1^)	*r* = 0.38 (*p* < 0.05)	*r* = 0.37 (*p* < 0.05)	*p* > 0.05	*r* = 0.40 (*p* < 0.05)	*r* = 0.36 (*p* < 0.05)	*r* = 0.50 (*p* < 0.001)
IL-10 (pg mL^− 1^)	*r* = − 0.36 (*p* < 0.001)	*r* = − 0.38 (*p* < 0.05)	*p* > 0.05	*r* = − 0.44 (*p* < 0.05)	*r* = − 0.34 (*p* < 0.05)	*r* = − 0.54 (*p* < 0.001)
TNF-α (pg mL^− 1^)	*r* = 0.43 (*p* < 0.001)	*r* = 0.41 (*p* < 0.05)	*p* > 0.05	*r* = 0.37 (*p* < 0.05)	*r* = 0.35 (*p* < 0.05)	*r* = 0.48 (*p* < 0.001)
INF-γ (pg mL^− 1^)	*r* = 0.47 (*p* < 0.001)	*r* = 0.44 (*p* < 0.001)	*p* > 0.05	*r* = 0.41 (*p* < 0.05)	*r* = 0.40 (*p* < 0.05)	*r* = 0.59 (*p* < 0.001)
PCO (nmol mg^− 1^ protein)	*r* = 0.46 (*p* < 0.001)	*r* = 0.45 (*p* < 0.001)	*p* > 0.05	*r* = 0.49 (*p* < 0.001)	*r* = 0.45 (*p* < 0.05)	*r* = 0.64 (*p* < 0.001)
GST (U GST mg^− 1^ protein)	*p* > 0.05	*p* > 0.05	*p* > 0.05	*r* = − 0.34 (*p* < 0.05)	*r* = − 0.34 (*p* < 0.05)	*r* = − 0.26 (*p* < 0.05)

*t,t-MA trans,trans*-muconic acid, *IL-1β* interleukin-1β, *IL-6* interleukin-6, *IL-10* interleukin-10, *TNF-α* tumor necrosis factor-α, *IFN-γ* interferon-γ, *PCO* protein carbonyl, *GST* glutathione S-transferase

Table 3 summarizes the correlations found among the evaluated biomarkers of effect. It was possible to observe significant correlations between immunological biomarkers and inflammatory biomarkers, immunological biomarkers and oxidative stress biomarkers, and oxidative stress biomarkers and inflammatory biomarkers. Furthermore, a negative correlation was observed between the oxidative stress biomarkers PCO levels and GST activity (*r* = − 0.31; *p* < 0.05).

**Table 3:** Correlations among biomarkers of effect (*n* = 66)

Immunological biomarkers vs. Inflammatory biomarkers					
	IL-1β (pg mL^− 1^)	IL-6 (pg mL^− 1^)	IL-10 (pg mL^− 1^)	TNF-α (pg mL^− 1^)	INF-γ (μg mL^− 1^)
CD80 monocyte expression (%)	*p* > 0.05	*p* > 0.05	*r* = 0.28 (*p* < 0.05)	*p* > 0.05	*r* = − 0.31 (*p* < 0.05)
CD86 monocyte expression (%)	*r* = − 0.27 (*p* < 0.05)	*r* = − 0.29 (*p* < 0.05)	*r* = 0.28 (*p* < 0.05)	*r* = − 0.35 (*p* < 0.05)	*r* = − 0.42 (*p* < 0.001)
Immunological biomarkers vs. Oxidative Stress biomarkers					
	PCO (nmol mg^− 1^ protein)			GST (U GST mg^− 1^ protein)	
CD80 monocyte expression (%)	*r* = − 0.41 (*p* < 0.001)			*p* > 0.05	
CD86 monocyte expression (%)	*r* = − 0.43 (*p* < 0.001)			*p* > 0.05	
Oxidative Stress biomarkers vs. Inflammatory biomarkers					
	IL-1β (pg mL^− 1^)	IL-6 (pg mL^− 1^)	IL-10 (pg mL^− 1^)	TNF-α (pg mL^− 1^)	INF-γ (pg mL^− 1^)
PCO (nmol mg^− 1^ protein)	*r* = 0.51 (*p* < 0.001)	*r* = 0.49 (*p* < 0.001)	*r* = − 0.42 (*p* < 0.001)	*r* = 0.46 (*p* < 0.001)	*r* = 0.54 (*p* < 0.001)
GST (U GST mg^− 1^ protein)	*r* = − 0.29 (*p* < 0.05)	*r* = − 0.28 (*p* < 0.05)	*p* > 0.05	*r* = − 0.35 (*p* < 0.05)	*r* = − 0.39 (*p* < 0.001)

*IL-1β* interleukin-1β, *IL-6* interleukin-6, *IL-10* interleukin-10, *TNF-α* tumor necrosis factor-α, *IFN-γ* interferon-γ, *PCO* protein carbonyl, *GST* glutathione S-transferase

Exposure time was positively correlated with urinary *t,t*-MA levels (*r* = 0.67; *p* < 0.001). Additional correlations between exposure time and immunological, inflammatory, and oxidative stress biomarkers are shown in Table 4.

**Table 4:** Correlations among exposure time and immunological, inflammatory, and oxidative stress biomarkers (*n* = 66)

	Exposure time (years)
CD80 monocyte expression (%)	*r* = − 0.34 (*p* < 0.05)
CD86 monocyte expression (%)	*r* = − 0.50 (*p* < 0.001)
IL-1β (pg mL^− 1^)	*r* = 0.57 (*p* < 0.001)
IL-6 (pg mL^− 1^)	*r* = 0.54 (*p* < 0.001)
IL-10 (pg mL^− 1^)	*r* = − 0.56 (*p* < 0.001)
TNF-α (pg mL^− 1^)	*r* = 0.53 (*p* < 0.001)
INF-γ (pg mL^− 1^)	*r* = 0.59 (*p* < 0.001)
PCO (nmol mg^− 1^ protein)	*r* = 0.58 (*p* < 0.001)
GST (U GST mg^− 1^ protein)	*r* = − 0.27 (*p* < 0.05)

*IL-1β* interleukin-1β, *IL-6* interleukin-6, *IL-10* interleukin-10, *TNF-α* tumor necrosis factor-α, *IFN-γ* interferon-γ, *PCO* protein carbonyl, *GST* glutathione S-transferase

Based on the correlations shown above, variables were selected to verify their influence on the increased pro-inflammatory biomarkers (IL-1β, IL-6, TNF-α, and IFN-γ). The multiple linear regression model, displayed in Table 5, accounted for approximately 58, 55, 58, and 63% of the increased pro-inflammatory cytokines IL-1β, IL-6, TNF-α, and IFN-γ, respectively. For all the inflammatory biomarkers, benzene exposure was the only factor that significantly contributed to the increased levels of the markers: IL-1β (β estimate = 0.437; *p* = 0.018); IL-6 (β estimate = 0.519; *p* = 0.007); TNF-α (β estimate = 0.496; *p* = 0.008); and IFN-γ (β estimate = 0.388; *p* = 0.031).

**Table 5:** Multiple linear regression models

	IL-1β (pg mL^− 1^)		IL-6 (pg mL^− 1^)		TNF-α (pg mL^− 1^)		INF-γ (pg mL^− 1^)	
	*R* square = 0.579		*R* square = 0.555		*R* square = 0.584		*R* square = 0.627	
	*Β*	*p*-values	*β*	*p*-values	*β*	*p*-values	*β*	*p*-values
Benzene (μg m^−3^)^a^	0.437	*p* < 0.05	0.519	*p* < 0.05	0.496	*p* < 0.05	0.388	*p* < 0.05
Toluene (μg m^−3^)^a^	0.213	*p* > 0.05	0.270	*p* > 0.05	0.221	*p* > 0.05	0.268	*p* > 0.05
*m-*Xylene (μg m^−3^)^a^	− 0.250	*p* > 0.05	− 0.182	*p* > 0.05	− 0.333	*p* > 0.05	− 0.080	*p* > 0.05
*p-*Xylene (μg m^− 3^)^a^	− 0.259	*p* > 0.05	− 0.285	*p* > 0.05	− 0.349	*p* > 0.05	− 0.177	*p* > 0.05
*t,t*-MA (μg g^− 1^ creatinine)^a^	− 0.319	*p* > 0.05	− 0.212	*p* > 0.05	−0.300	*p* > 0.05	−0.058	*p* > 0.05
CD86 monocyte expression (%)^a^	−0.404	*p* > 0.05	−0.381	*p* > 0.05	−0.428	*p* > 0.05	−0.374	*p* > 0.05
PCO (nmol mg^−1^ protein)^a^	0.140	*p* > 0.05	0.017	*p* > 0.05	0.051	*p* > 0.05	0.053	*p* > 0.05
GST (U GST mg^−1^ protein)^a^	−0.165	*p* > 0.05	−0.071	*p* > 0.05	−0.196	*p* > 0.05	−0.155	*p* > 0.05
Exposure time (years)^a^	−0.463	*p* > 0.05	−0.481	*p* > 0.05	−0.461	*p* > 0.05	−0.387	*p* > 0.05
Age (years)^a^	0.277	*p* > 0.05	0.229	*p* > 0.05	0.148	*p* > 0.05	0.161	*p* > 0.05
Smoking^b^	−0.129	*p* > 0.05	−0.087	*p* > 0.05	−0.085	*p* > 0.05	−0.123	*p* > 0.05

*t,t-MA trans,trans*-muconic acid, *IL-1β* interleukin-1β, *IL-6* interleukin-6, *TNF-α* tumor necrosis factor-α, *IFN-γ* interferon-γ, *PCO* protein carbonyl, *GST* glutathione S-transferase

^a^Categorical variable

^b^Continuous variable

## Discussion

In this study, we observed the influence of BTX compound exposure on the immunological, inflammatory, and oxidative stress biomarkers evaluated. Furthermore, the data suggest that the immunological and inflammatory alterations observed in this study are interrelated, and connected to the imbalance of the oxidative status.

Personal occupational exposure to BTX, shown in our study, characterizes low-level exposure to these aromatic hydrocarbons, since all the found values were below the Threshold Limit Value-Time Weighted Average (TLV-TWA) proposed by ACGIH for working environments. However, even so, it was possible to observe significantly higher levels of benzene, toluene, and xylene exposure in the GSA group compared to the non-exposed group. These findings are in accordance with previous studies that have also found airborne BTX concentrations lower than the ACGIH limits [2–4, 11, 17]. Additionally, the strong correlations shown between airborne BTX concentrations evidenced that the GSA group was exposed to a mixture of multiple organic solvents.

According to Carrieri et al. [3], urinary *t,t*-MA is a widely used biomarker in routine practice for the biological monitoring of benzene exposure, mainly because of the simple analytical method used for its determination, easily available in most industrial and environmental toxicology laboratories. However, diet and smoking interference in this biomarker of exposure may compromise its specificity. Despite that, in our study, *t,t*-MA was confirmed as a good indicator of exposure to low levels of benzene exposure, since there were significantly elevated levels in the GSA group in relation to the non-exposed group. In addition, *t,t*-MA excretion correlated with personal benzene exposure, which points to the relevance of this biomarker of exposure, even at reduced levels. The same cannot be visualized for the urinary biomarker of exposure to toluene, since HA did not show any significant difference between the groups, nor was it correlated with personal toluene exposure, as previously shown in studies by our group [4, 17]. Also, m-HA, urinary biomarker of xylene exposure, did not prove to be a good marker.

The alterations observed in the immunological biomarkers evaluated in our study (CD80 and CD86 expression in monocytes) are in accordance with previously described data [2, 4, 17]. Furthermore, the univariate correlations showed that the reduced expressions of CD80 and CD86 were related to BTX exposure, proving the interference of these chemical compounds in the immune system of occupationally exposed individuals.

The co-stimulatory molecules CD80 and CD86 are recognized as the main co-stimulatory molecules of the immune system [17], and are considered one of the most important signaling mechanisms and regulators of adaptive cellular immunity [23, 24]. The absence or depletion of these co-stimulatory molecules results in hyporresponsiveness, development of anergic T cells, or inefficient immune surveillance [17, 25, 26]. According to Sauer et al. [17], immunological alterations, as observed in our study, could contribute to the development of benzene immunocarcinogenic effects, since it is known that the immune system is highly involved in carcinogenesis.

The GSA group evaluated in our study presented increased pro-inflammatory cytokines (IL-1β, IL-6, TNF-α, and IFN-γ), accompanied by decreased levels of the anti-inflammatory IL-10 cytokine, which is in accordance with previous literature [27]. Although the lower expression of the CD80 and CD86 adhesion molecules are linked to an ineffective immune response, and consequently, to a compromised inflammatory response, studies have shown that chronic exposure to toxic agents, such as BTX compounds, could contribute to the development of an inflammatory process, which is involved in the development of different types of cancer and tumor progress [28, 29]. In addition, during the hepatic biotransformation of BTX compounds, many oxidative and reactive species are produced, which could contribute to the increased of pro-inflammatory cytokines, as evidenced in our results through of moderate correlations among immunological and inflammation biomarkers. CD86 expression in monocytes was positively correlated with IL-10 levels and negatively associated with pro-inflammatory biomarkers. These data suggest an immunological impairment scenario, accompanied by an inflammatory process, which could contribute to carcinogenesis, as described above.

According Spatari et al. [30], aromatic hydrocarbons found in fuel are active agents responsible for suppressing immune response. In our study, according to the multiple linear regression model, personal benzene exposure was the only variable that significantly contributed to the increased levels of the pro-inflammatory biomarkers (IL-1β, IL-6, TNF-α, and IFN-γ), These findings suggested that the immunological and inflammatory alterations observed in our study could be related to carcinogenic process development due to benzene exposure. However, additional and further studies are needed to confirm this hypothesis.

The immunological and inflammatory alterations observed in our study are connected to the oxidative stress biomarkers, which could be shown by the univariate correlations. Additionally, the GSA group showed increased levels of PCO accompanied by decreased GST activity when compared to the non-exposed group. Furthermore, these alterations were linked to BTX exposure, confirming the involvement of these xenobiotics in the imbalance of the oxidative status observed.

According to Barreto et al. [31], during the biotransformation of benzene, multiple forms of reactive species are produced, which could contribute to the development of oxidative damage, and could be the cause of increased levels of the biomarkers of protein damage observed in our study. Besides oxidative damage to protein, the continuous production of reactive species during benzene metabolism could induce toxicity in key cellular components, such as DNA [32]. Indeed, the activation of benzene and its metabolites culminates in damage to lipids, proteins, and DNA carbohydrates through various chemical reactions, leading to functional alterations in different tissues [33]. Furthermore, chronic benzene exposure compromises antioxidant capacity, which also contributes to the development of oxidative damage in exposed individuals [10, 34].

All the immunological, inflammatory, and oxidative stress biomarkers evaluated were correlated with exposure time. These findings indicate the possibility of using these biomarkers in the continuous monitoring of occupational hazards.

## Conclusions

The main findings of this study are related to the fact that all the alterations evidenced could be involved in the damage caused by occupational benzene exposure, especially the inflammatory process, which had not yet been detected in previous studies of our group, involving individuals occupationally exposed to benzene. Additional studies should be performed to elucidate the potential involvement of each biomarker. However, the continuous monitoring of these biomarkers could contribute to the early detection of the damages caused by benzene.

Taken together, these findings showed the influence of exposure to BTX compounds, especially benzene, on the immunological, inflammatory, and oxidative stress biomarkers evaluated. Furthermore, the data suggest a relationship among the evaluated biomarkers of effect, which, if continuously monitored, could contribute to providing early signs of damage to biomolecules in subjects that are occupationally exposed to BTX compounds. Additional measures, such as antioxidant intake, exposure elimination, also could contribute to the improvement of the life quality of occupational exposed subjects.

## Data Availability

The datasets used and/or analyzed during this study are available from the corresponding author on reasonable request.

## Abbreviations

ACGIH: American Conference of Governmental Industrial Hygienists
BEI: Biological exposure index
BTX: Benzene, toluene, and xylene
EDTA: Ethylenediamine tetra-acetic acid
GC-FID: Gas chromatographic - flame ionization detector
GSA: Gasoline station attendants
GST: Glutathione S-transferase
HA: Hippuric acid
HPLC-UV: High-performance liquid chromatography with UV detection
IFN-γ: Interferon-γ
IL-10: Interleukin-10
IL-1β: Interleukin-1β
IL-6: Interleukin-6
mHA: Methylhippuric acid
NE: Non-exposed
PCO: Protein carbonyl
SEM: Standard error of the mean
SPE: Solid phase extraction
*t,t*-MA: *trans,trans*-muconic acid
TLV-TWA: Threshold Limit Value-Time Weighted Average
TNF-α: Tumor necrosis factor-α
